# Pulmonary Atresia with Intact Ventricular Septum, an Evolving Strategy in the Era of PDA Stenting: Single Center Experience

**DOI:** 10.1007/s00246-025-03769-w

**Published:** 2025-01-22

**Authors:** Kamel Shibbani, John Nigro, Rohit Rao, Brent M. Gordon, Henri Justino, Laith AlShawabkeh, Howaida El-Said

**Affiliations:** 1 Department of Pediatrics, Section of Cardiology, Rady Children’s Hospital, University of California-San Diego, San Diego, CA USA; 2 Division of Cardiology, Department of Pediatric, Stead Family Children’s Hospital, University of Iowa, Iowa City, USA; 3 Department of Cardiology, ACHD, Rady Children’s Hospital, University of California-San Diego, San Diego, CA USA

**Keywords:** Pulmonary atresia intact ventricular septum, PDA stenting, RV decompression, RV dependent coronary circulation

## Abstract

Repair or palliation of pulmonary atresia with intact ventricular septum (PA/IVS) often falls into one of 4 categories: cardiac transplant, 2-ventricular circulation, 1.5 ventricle circulation, or single ventricle circulation. The optimal management strategy has been an area of much debate. We sought to review the management strategy of patients with PA/IVS at our institution to better understand what metrics can be used to guide management and initial interventions. The study aims to examine the outcomes of a single-center approach to managing patients with PA/IVS. Our cohort included 29 patients; one patient underwent a planned transplant at ten days of life (3.4%), 12 underwent repair via a two-ventricle circulation (41.4%), 7 underwent repair with 1.5 ventricle circulation (24.1%), and 7 underwent repair with single ventricle circulation (24.1%). Survival was achieved in 93.1% with two patients (6.9%) expiring. The TV annulus z-score was significantly different between the three groups, with the 1 V group having the smallest median TV annulus *z*-score at − 4.04 (IQR − 4.60– − 3.60) and the 2 V group having the largest median TV *z*-score at − 1.4 (IQR − 2.24– − 0.12). Six patients underwent late right ventricular decompression. We present a post-hoc algorithm to help guide treatment strategies for patients with PA/IVS.

## Introduction

Repair or palliation of pulmonary atresia with intact ventricular septum (PA/IVS) falls into one of 4 categories: cardiac transplant, 2-ventricular circulation, 1.5 ventricle circulation, or single ventricle circulation. The optimal management strategy is an area of much debate. There is little disagreement at the extreme ends of the PA/IVS spectrum. Patients with two adequately sized ventricles with no evidence of right ventricular-dependent coronary circulation (RVDCC) are considered suitable candidates for 2-ventricular circulation. In contrast, those with diminutive right ventricles and those with RVDCC often undergo transplant or single ventricle circulation. However, managing patients with an anatomy that falls between these extremes must be clarified. Even the definitions of an adequately sized right ventricle or RVDCC are subject to disagreement. The tricuspid valve annulus z-score has been suggested as a surrogate for determining the adequacy of the right ventricle. However, the literature has many papers suggesting various cutoffs for such a determination ranging from − 5.68 to − 2.8 [1–4].

Interestingly, even converting an absolute tricuspid valve annulus diameter in mm to a z-score is subject to significant variability, depending on the data set used [5]. Furthermore, the definition of RVDCC is not universal, with some authors positing that the involvement of two or more coronary arteries through stenosis or ostial atresia is required to make this diagnosis [6, 7]. In contrast, others argue that any stenosis or atresia proximal to a fistula between the coronary artery and the right ventricle constitutes RVDCC [8]. Others, too, argue that the presence of significant fistulas, even in the absence of any coronary artery stenosis or atresia, is enough to compromise coronary flow and cause RVDCC [9]. Considering the variability in diagnosing and managing PA/IVS, as well as the historically high mortality rate of these patients of up to 54% [4, 10], we sought to review the management strategy of patients with PA/IVS at our institution in hopes of better understanding what metrics can be used to guide surgical management and initial interventions. The study aims to examine the outcomes of a single-institution approach to managing patients with PA/IVS and provide a post-hoc algorithm we currently utilize to achieve this outcome.

## Methods

This single-center retrospective cohort study was performed at Rady Children’s Hospital. The normality of the data were assessed using the Shapiro–Wilk test. Comparison between groups was done using the Kruskal–Wallis H test. All patients born with a diagnosis of PA/IVS between 1/1/2013 and 6/30/2023 were included in the study. The variables collected included sex, gestational age at birth, weight at birth, weight at surgery, age at first intervention, type of first intervention, TV annulus (absolute and z-score), PV annulus (absolute and *z*-score), type of pulmonary atresia, presence of RV to coronary fistulas, presence of coronary stenosis or atresia, morphology of the RV (unipartite, bipartite, or tripartite), type of surgical repair, re-intervention (defined as the need to perform a procedure outside of initial pulmonary valvuloplasty, initial PDA stent or central shunt, and surgical palliation or repair) and survival. Outcomes included transplant, single ventricle circulation, 1.5 ventricle/pulsatile Glenn circulation, or two-ventricle circulation. Two-ventricle circulation is achieved when systemic venous blood returns to the right atrium and is delivered to the pulmonary artery through the right ventricle without a significant residual right-to-left shunt at the atrial-level. Single ventricle circulation is defined as a Fontan circulation, whereas 1.5 ventricle circulation is defined as a bidirectional Glenn anastomosis in the setting forward flow from the right ventricular outflow tract (RVOT) into the PA. Neonatal RV decompression was avoided if RVDCC could not be definitively ruled out or if the right-sided structures were deemed to be too diminutive to support 2V repair (unipartite RV or TV annulus z-score < − 4). The Boston Children’s Hospital z-score system was used to convert absolute TV annulus measurement to *z*-score [11]. The primary outcome assessed was survival.

## Results

### Demographics

A total of 29 patients were included in the study, with 27 (93.1%) alive at the last available follow-up. The median follow-up duration was five years (IQR 2.3–6.3 years). Two patients were diagnosed with pulmonary atresia with intact ventricular septum (PA/IVS) with dysplastic tricuspid valves and severe tricuspid valve regurgitation.

The median gestational age for the cohort was 38.43 weeks (IQR 36.14–39.28 weeks), with a birth weight of 3,330 g (IQR 2822–3650 g). The median weight at the initial surgery or intervention was 3,331 g (IQR 2822–3650 g). The median age at the first intervention was five days (IQR 2–8 days). The cohort's median tricuspid valve (TV) annulus z-score was − 2.67 (IQR − 3.54 to − 1.63).

Among the 29 patients included in the study, 23 (79.3%) had their initial pulmonary blood flow established by placing a patent ductus arteriosus (PDA) stent. In contrast, five patients (17.2%) achieved pulmonary blood flow via an aortopulmonary shunt. The remaining patient, who underwent a neonatal heart transplant, was maintained on prostaglandin E (PGE) infusion and successfully received the transplant within the first two weeks of life. The decision of PDA stent vs an aortopulmonary shunt was predomintanly era-specific, with early experience favoring mBTT shunt placement (55% of the patients in the first 4 years of the study underwent mBTT shunt placement) and later experience favouring PDA stenting (94% of the patiented in the last 6 years of the study underwent PDA stent placement).

Regarding the pulmonary valve interventions, 19 patients underwent catheterization procedures that included opening the pulmonary valve and placing a PDA stent. Notably, 5 of these patients initially had a procedure limited to pulmonary valve opening without PDA stenting. These patients subsequently required PDA stenting, performed as a separate procedure 1 to 4 days after the initial valvuloplasty.

Of the 19 patients who underwent pulmonary valvuloplasty and RV decompression, 12 (63.2%) had the pulmonary valve crossed using radiofrequency energy, and 7 (36.8%) had the pulmonary valve crossed using a wire.

When comparing the three surgical groups, a significant difference was observed in the age at first intervention, with patients in the 2 V circulation group being the youngest at the time of their initial procedure. Additionally, the tricuspid valve (TV) annulus z-score varied significantly among the groups; the 2 V circulation group had the largest median TV *z*-score, while the 1 V circulation group had the smallest (− 1.4 vs − 4.04). There were no statistically significant differences between the groups regarding birth weight, weight at first intervention, or gestational age at birth (Table 1). The prevalence of right ventricle to coronary artery fistulas was 25% in the 2 V circulation group and 85.7% in the 1 V circulation group.

**Table 1:** Characteristics of patients with PA/IVS grouped by surgical outcome (excluding the 2 deaths and 1 transplant patient)

		1 V	1.5 V	2 V	*p*-value
*n*	Count (%)	7 (24.1)	7 (24.1)	12 (41.4)	
	Male	6	4	6	
Median (IQR)	GA at birth (weeks)	37.00 (35.07–39.07)	38.43 (37.29–39.07)	38.79 (37.42–39.32)	NS
	Age at 1st intervention (days)	6 (4.5–13)	5 (4–7.5)	2 (2–4.5)	0.03
	Weight at birth (grams)	3330 (2450–3585)	2940 (2650–3120)	3502 (2939–3665)	NS
	Weight at surgery (grams)	3330 (2980–3560)	2900 (2700–3090)	3512 (3048–3855)	NS
	TV annulus size (mm)	4.3 (4.3–4.7)	6.0 (5.8–7.0)	8.7 (6.9–11.3)	0.0007
	TV annulus *Z*-score	− 4.04 (− 4.60– − 3.60)	− 2.64 (− 2.80– − 2.50)	− 1.4 (− 2.24– − 0.12)	0.0009
	Pulmonary valve size (mm)	2.85 (2.2–3.25)	NA	4.9 (4.3–5.4)	0.039
	Pulmonary valve *Z*-score	− 3.5 (− 4.3– − 2.1)	NA	− 2.6 (− 3.2– − 2.4)	NS

### Primary Outcome

In a cohort of 29 patients, one underwent a neonatal heart transplant. The remaining patients received palliation through one of three approaches: two-ventricle circulation (2 V), one-and-a-half ventricle circulation (1.5 V), or single ventricle circulation (1 V). Specifically, 12 patients (41.4%) underwent 2 V circulation, seven patients (24.1%) underwent 1.5 V circulation, and another seven patients (24.1%) underwent 1 V circulation.

**Fig. 1:**
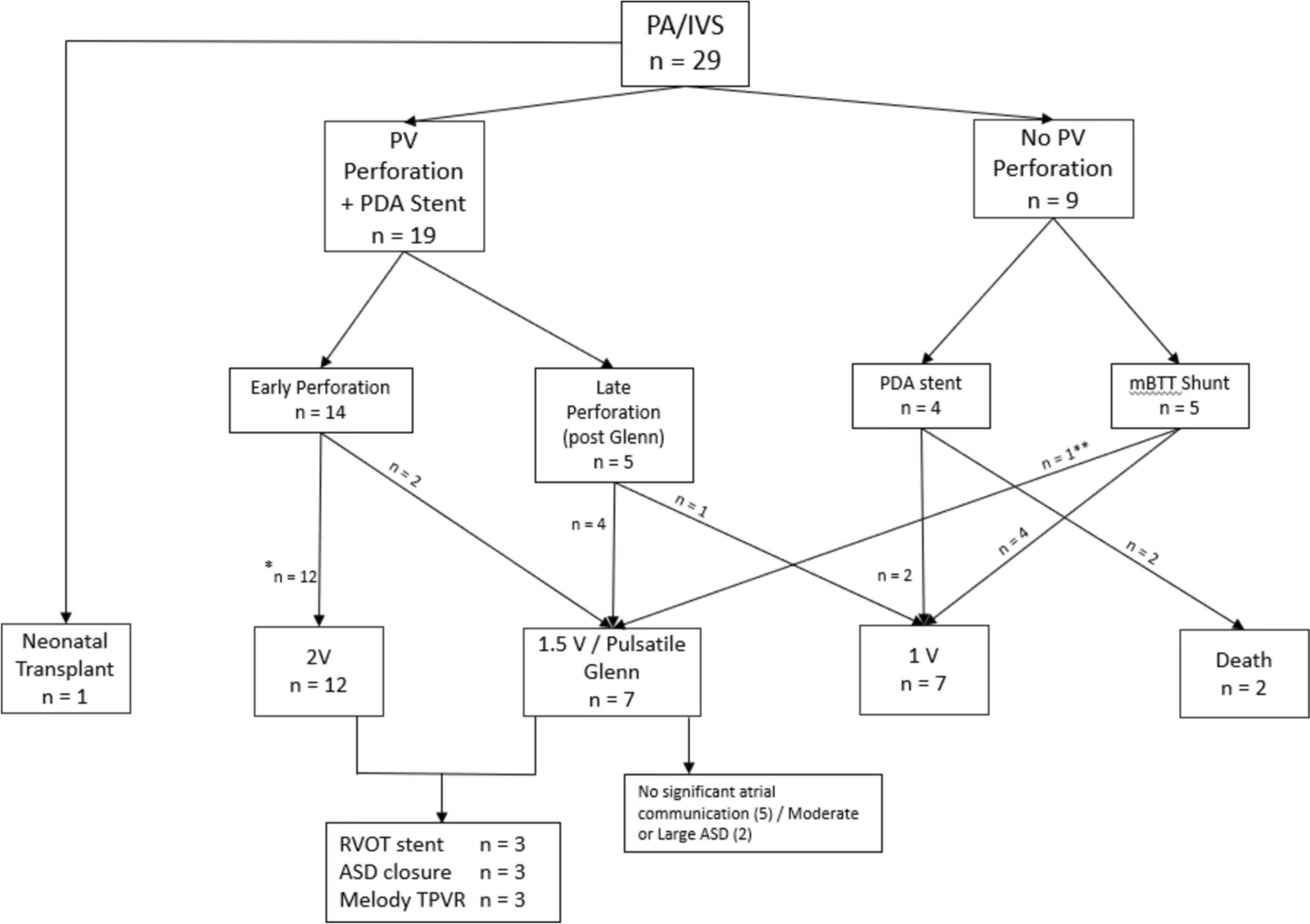
Summary of patient outcome. *PV* pulmonary valve, *PDA* patent ductus arteriosus, *mBTT shunt* modified blalock taussig thomas shunt, *2V* two-ventricle repair, *1.5 V* one-and-a-half ventricle circulation, *1V* single ventricle circulation, *RVOT* right ventricular outflow tract, *ASD* atrial septal defect, *TPVR* transcatheter pulmonary valve replacement. *2 patients underwent surgical patching of the RVOT. **Late RV decompression with RV to PA conduit

Among these patients, 14 (48.3%) received right ventricular (RV) decompression via balloon valvuloplasty shortly after birth with 12 completing 2 V circulation and 2 requiring 1.5 V circulation. Of the remaining 15 patients who did not undergo early RV decompression, 6 underwent late decompression at an older age with five completing a 1.5 V circulation (two patients continue to have a moderate atrial-level communication). Seven patients achieved 1 V circulation, and two patients died (Fig. 1).

Of the 29 patients in the cohort, two patients (6.9%) died during follow-up, and one (3.4%) underwent a neonatal transplant. The transplanted patient presented with elevated troponin levels, decreased biventricular function on echocardiography, and proximal stenosis of the right coronary artery on angiography. He was listed for a transplant at seven days of life and received an orthotopic heart transplant at ten days of life.

The two patients who died both initially underwent ductal stenting followed by a bidirectional Glenn procedure. Neither of them had RV decompression or pulmonary valvuloplasty at any point. The first patient had pulmonary atresia with intact ventricular septum (PA/IVS), RVDCC, and aortic valve stenosis. This patient underwent PDA stenting and aortic valvuloplasty at ten days of life. At around five months, he was transferred to another hospital for a transplant due to decreased left ventricular function and worsening aortic valve stenosis. Instead of a transplant, the receiving hospital performed a Glenn procedure, and the patient died one month later.

The second patient had PA/IVS with a dysplastic tricuspid valve and severe tricuspid regurgitation alongside severe neonatal hypoxic-ischemic encephalopathy (HIE). Due to these comorbidities, ductal stenting was delayed until day 50 of life. He subsequently underwent a bidirectional Glenn at day 74 due to persistent desaturation and PDA stent fracture. His postoperative course was complicated by persistent hypoxemia requiring VA ECMO, and the parents elected to withdraw care on day 82.

### Early RV Decompression

Right ventricular (RV) decompression was performed in 20 patients, with 14 undergoing the procedure during the neonatal period, 5 undergoing the procedure past the neonatal period via pulmonary valvuloplasty, and 1 patient undergoing the procedure past the neonatal period via RV-PA conduit (Fig. 1). Of the 14 that underwent neonatal RV decompression, 12 patients proceeded to a two-ventricle (2V) circulation, while two underwent one-and-a-half ventricle (1.5V) circulation.

The two patients requiring 1.5V circulation had a borderline tricuspid valve annulus *z*-score at birth, measuring − 1.9 and − 2.8, respectively. One of these patients developed a cerebral abscess, partially attributed to the presence of an atrial-level communication, necessitating atrial septal defect (ASD) closure. During a catheterization procedure, test occlusion of the ASD revealed significant right atrial (RA) pressure elevation despite interval RV growth. Consequently, the patient was referred for a bidirectional Glenn procedure with ASD closure to offload the RV and prevent elevated filling pressures.

The second patient, despite RV decompression, continued to exhibit sub-pulmonary stenosis and elevated RV pressures. The RV did not grow adequately, and when further intervention was required, the RV was deemed too small to accommodate a 2V circulation. As a result, the patient underwent a bidirectional Glenn procedure with an RV outflow tract (RVOT) patch, ASD closure, and PDA takedown.

### Late RV Decompression

Among the 20 patients who underwent RV decompression, 6 underwent the procedure at a later stage. Five patients received late decompression through radiofrequency (RF) perforation and pulmonary valvuloplasty, while one patient underwent surgical placement of a right ventricle to pulmonary artery (RV-PA) conduit during the Glenn procedure (Fig. 1). The median age for late RV decompression was 24.4 months (IQR 20.7–42.6 months). The initial median tricuspid valve (TV) annulus z-score for this group was − 3.47 (IQR − 4.48 to − 2.6), with the smallest TV annulus *z*-score being − 5 in the patient who ultimately required single ventricle (1V) circulation (Table 2).

**Table 2:** Characteristics of patients undergoing late RV decompression. RVDCC = Right ventricle-dependent coronary circulation

		Late decompression	Reason for avoiding early RV decompression
*n*	Count	*6	Hypoplastic RV + small TV (*n* = 2) Avoiding a circular shunt in a patient with PA/IVS with severe tricuspid regurgitation (*n *= 1)
	Male	4	
Median (IQR)	GA at birth (weeks)	38.7 (36.74–39.10)	
	Age at 1st intervention (days)	6 (4.25–7.75)	
	Weight at birth (grams)	3183 (2964–3420)	
	Age at RV decompression (days)	743 (630–1295)	
	Initial TV-Annulus *Z*-score	− 3.47 (− 4.48– − 2.61)	

^*^5 late pulmonary valve perforation, 1 late surgical conduit

All five patients who underwent late RV decompression via RF perforation and pulmonary valvuloplasty had previously received PDA stenting to establish pulmonary blood flow, followed by a bidirectional Glenn procedure before the late opening of the pulmonary valve. The patient who underwent late RV decompression through surgical RV-PA conduit placement had PA/IVS with severe tricuspid valve regurgitation. Neonatal pulmonary valve perforation was avoided to prevent creating a circular shunt; instead, an aortopulmonary shunt was initially placed. At the time of the bidirectional Glenn procedure, the patient underwent tricuspid valve repair with surgical RV decompression via an RV to PA conduit.

Five of these six patients continued with 1.5V circulation, while one patient underwent 1V circulation. This patient experienced symptoms of hypoxemia and shortness of breath at two years old, prompting RV decompression to increase pulmonary blood flow. Despite the procedure, symptoms persisted, leading to the decision to pursue 1V circulation at three years of age. Note that of the seven patients who underwent a 1.5 V circulation, two continued to have a moderate atrial-level communication. The final state of these two patients (1.5V circulation vs 1V circulation) will be dictated by their ability to tolerate ASD closure.

**Fig. 2:**
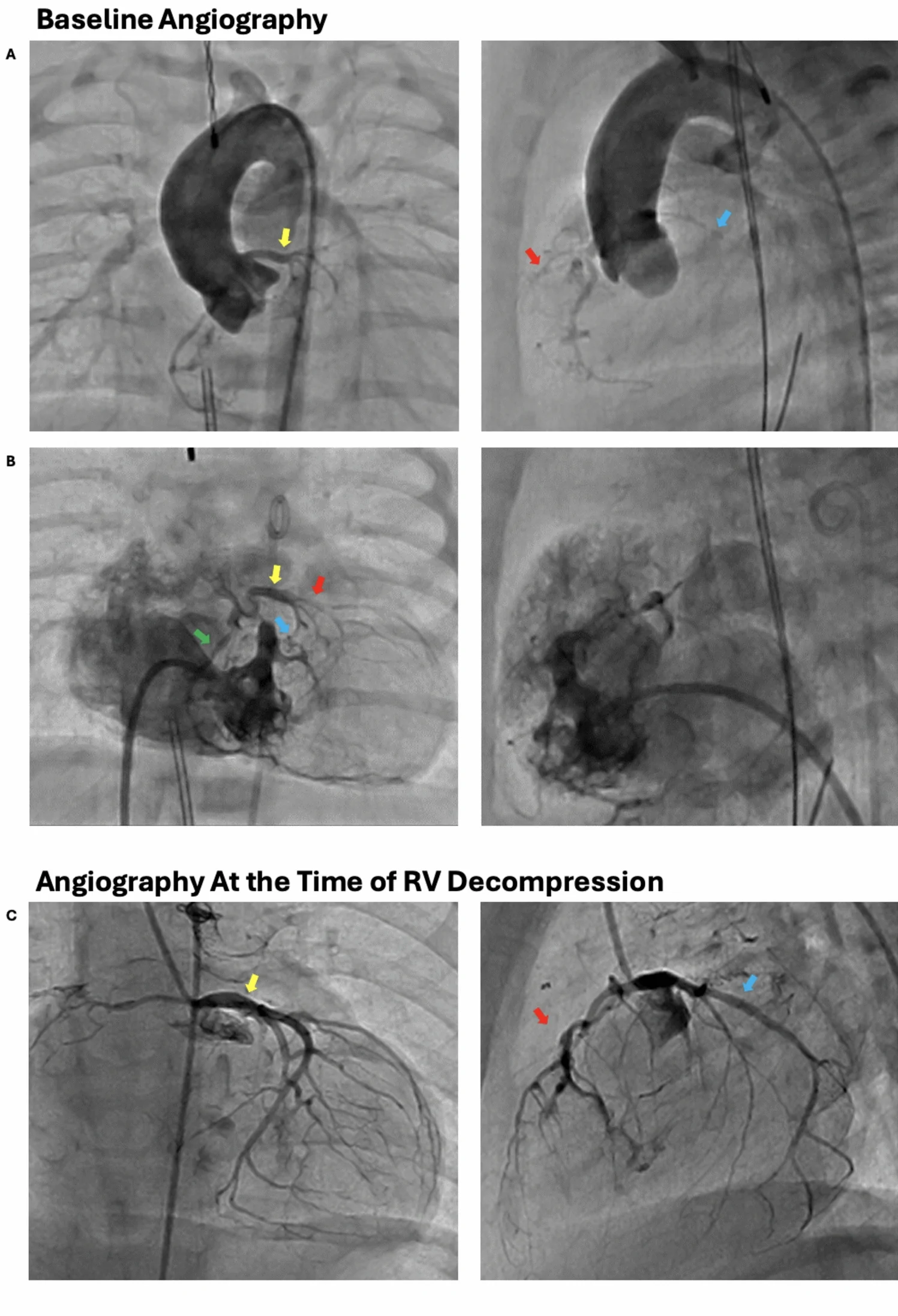
Comparison of coronary angiography at baseline and before late RV decompression in patient A. **A** Baseline coronary angiography concerning for RVDCC with poor filling of the LAD and circumflex artery. Yellow arrow indicates the LMCA, with very faint filling of the LAD and circumflex. Red arrow indicates the LAD. Blue arrow indicates the circumflex. **B** Retrograde filling of the coronary arteries noted with RV angiography of the same patient. Yellow arrow indicates the LMCA, Red arrow indicates the LAD. Blue arrow indicates the circumflex. Green arrow indicates the RCA. **C** Angiography at the time of RV decompression of the same patient showing normal filling of the left coronary system. Yellow arrow indicates the LMCA, with robust filling of the LAD and circumflex. Red arrow indicates the LAD. Blue arrow indicates the circumflex

**Fig. 3:**
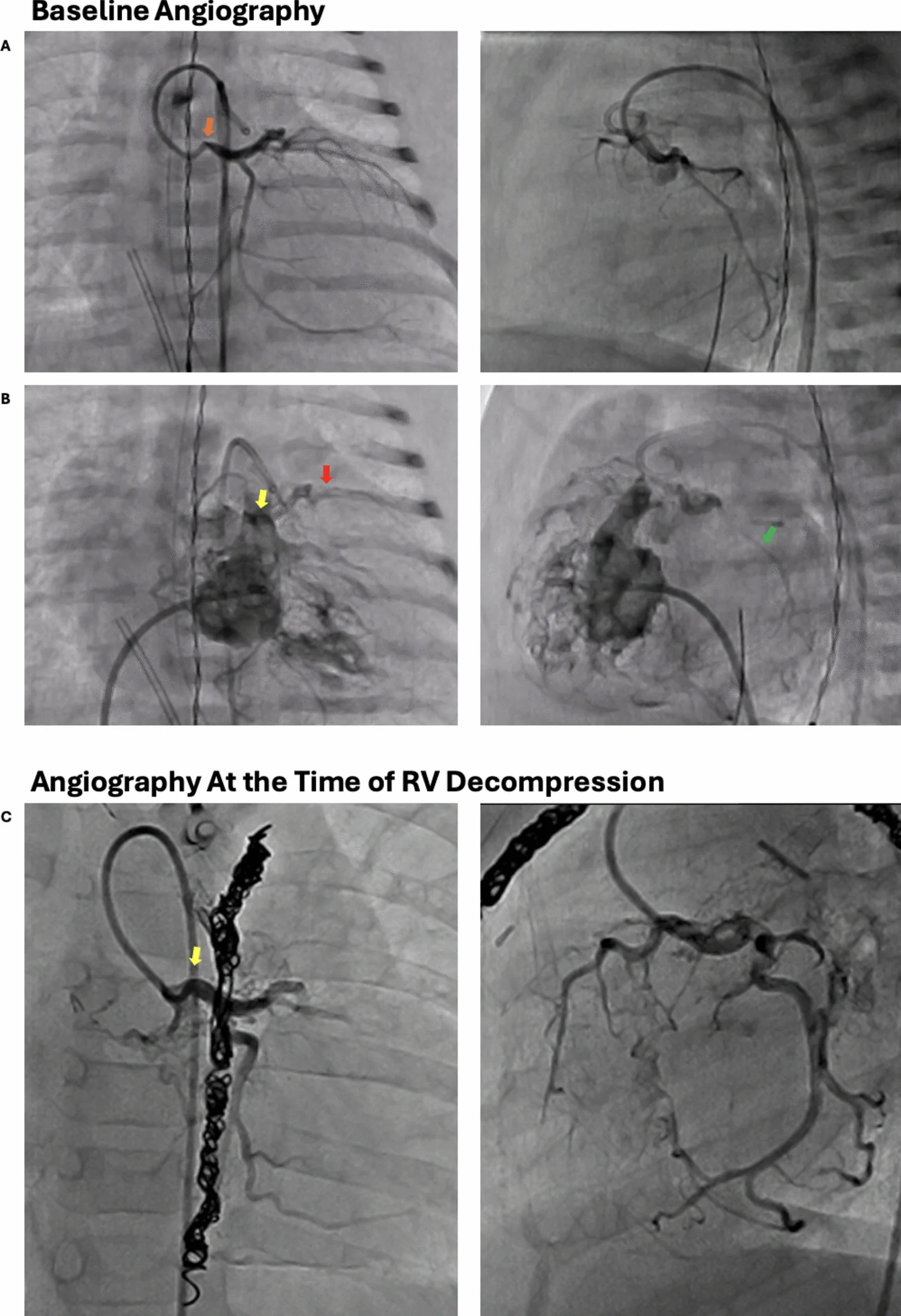
Comparison of coronary angiography at baseline and prior to late RV decompression in patient B. **A** Baseline coronary angiography concerning for narrowing of the proximal LMCA. Orange arrow shows narrow ostium of the LMCA. **B** Baseline RV angiography showing retrograde filling of the left coronary system. Yellow arrow indicates LMCA. Red arrow indicates LAD. Blue arrow indicates circumflex. **C** Angiography at the time of RV decompression of the same patient that showed no LMCA narrowing (arrow)

Before late decompression, all five patients underwent extensive evaluations, including coronary reassessment via angiography and RV size and function reassessment through echocardiography and/or MRI. None of the patients showed evidence of RV-dependent coronary circulation (RVDCC) before late RV decompression. Initially, in three patients, RVDCC could not be definitively ruled out on angiography, but subsequent studies revealed coronary arteries without RVDCC, allowing for RV decompression (Figs. 2 and 3).

### Interstage Procedures

Among the 19 patients who underwent either 2V or 1.5V circulation, 14 required re-intervention before the final surgical procedure. Specifically, four patients underwent balloon angioplasty of the PDA stent, another four required re-stenting of the PDA, and two patients needed surgical RV outflow tract (RVOT) patch placement. Additionally, one patient had balloon angioplasty of the PDA stent and subsequent re-stenting.

Various other procedures were also performed, with ten patients undergoing more than one re-intervention. These included atrial septal defect (ASD) device closure in three patients, RVOT stenting in three patients, transcatheter pulmonary valve (Melody valve) placement in two patients and repeat balloon pulmonary valvuloplasty in seven patients.

## Discussion

Our study assessed the outcomes of patients with PA/IVS in our institution. Our cohort included 29 patients; one had a neonatal transplant – 41.4% underwent two-ventricle circulation, 24.1% underwent 1.5 ventricle circulation, and 24.1% underwent single ventricle circulation. Survival was achieved in 93.1%. A multicenter study examining surgical outcomes of PA/IVS from 10 different institutions cited a wide range of 2V circulation between 8 and 56% [4], which places our 41.4% 2V circulation rate at the higher end of the spectrum. One study suggests that the combination of PA/IVS with Ebstein anomaly predisposes patients to a higher risk of death and inability to achieve 2V circulation [4]. In our cohort, one of the two mortalities was diagnosed with a dysplastic tricuspid valve with severe TR in the setting of a diminutive RV, which mimics some of the physiological features of Ebstein anomaly. The fact that this patient also had HIE possibly increased the likelihood of Glenn failure. Studies have shown that low preoperative SVC flow is associated with Glenn failure [12] and that perinatal neurological insult is associated with low SVC flow [13]. The other patient that did not survive had aortic stenosis in addition to PA/IVS, a rare combination of defects that complicates post-natal care, with two reports in the literature pointing to poor outcomes in these patients [14], 15.

Consistent with much of the literature, our data point towards a correlation between tricuspid valve z-score and propensity to undergo 2V vs 1.5V vs 1V circulation [1, 2. Patients who underwent 1V circulation had the lowest median TV *z*-score at − 4.04. Although multiple studies have shown an association between tricuspid valve z-score and propensity to undergo a biventricular repair [2], 4, 17, there continues to be no agreement on the appropriate z-score cut-off. This debate is complicated by the presence of different data sets to convert an absolute TV annulus measurement to a *z*-score, with the most frequently used being the Daubney *z*-score data set and the Rowlatt *z*-score data set. The Daubney data set is derived from echocardiographic measurements, whereas the Rowlatt data set is based on cadaveric measurements [18], 19. This lack of uniformity means an identical TV annulus measurement can have significantly different z-scores depending on the data set [5]. The TV *z*-score for patients undergoing 2V circulation in our cohort skews on the more conservative side compared to the literature, with a median of − 0.89. However, given the variability in the data sets used, it is challenging to reach conclusions by comparing our median *z*-score and published literature.

We had 19 patients who underwent pulmonary valvuloplasty and PDA stenting, with 5 of those initially undergoing pulmonary valvuloplasty without PDA stenting. All five patients subsequently underwent PDA stenting as a separate procedure. Patent ductus arteriosus stenting in patients with PA/IVS is associated with decreased hospital stay and days in the intensive care unit [20] with no difference in mortality or surgical outcome (2V vs 1.5 V vs 1V) [16]. Importantly, patients without a PDA stent had a significantly higher incidence of early re-intervention, with no re-intervention reported in patients undergoing PDA stenting and no increase in comorbidities in the group undergoing PDA stenting [16]. Our data are consistent with the literature and suggests that in patients who are planning to undergo RV decompression, either with the hope of undergoing 1.5V or 2V circulation, it is prudent to consider simultaneous PDA stenting, which is our institutional approach. It is worth noting that if the PDA is deemed too large to deploy a PDA stent at the time of valvuloplasty safely, a patient could require a repeat procedure for PDA stenting after discontinuation of prostaglandins to allow for the PDA to constrict enough to land a stent safely.

Six of our patients underwent late decompression, with 5 undergoing pulmonary valve perforation post-Glenn and one patient undergoing RV-PA conduit at the time of Glenn. The initial evaluation of these patients was either concerning for RVDCC or showed a diminutive right heart that was thought to be only compatible with 1V palliation. As a result, these patients were initially placed in the 1V pathway. Later catheterizations revealed no obvious RVDCC, and the patients subsequently underwent RV decompression and palliation through a 1.5V. Eighty-three percent of them tolerated the 1.5V circulation very well. Note that two patients continue to have a moderate or large atrial-level communication, which means that the final determination on which type of circulation these patients will have (1.5V vs 1V) will be dependent on how well they tolerate the closure of the atrial-level communication. There are no reports in the literature about late RV decompression. Patients have typically been directed and committed towards one surgical pathway at the neonatal stage with minimal subsequent reassessment of candidacy for other pathways. Our data suggest that reassessment of candidacy for other pathways should be an ongoing process, with some patients undergoing a 1.5V circulation after initially being identified as having anatomy suitable only for 1V circulation. This idea of late RV decompression and ongoing assessment allows for a more significant margin of safety in instances where RVDCC cannot be entirely ruled out. Our data suggests that these patients do not have to be committed to a 1V circulation early and could safely be palliated with a 1.5V circulation. It is worth noting that the median age at RV decompression in our cohort was 743 days. This was due to a change in approach at our institution, as opposed to an attempt to intentionally delay decompression. Indeed, it is our strategy to proceed with RV decompression as soon as RVDCC can be safely ruled out to allow for maximum potential RV growth. Still, the experience presented here shows that RV decompression can be safely done at older ages. This highlights that patients who have gone down a 1V circulation route need not be committed to that route if subsequent assessment shows no evidence of RVDCC, even if that assessment is done at an older age.

**Fig. 4:**
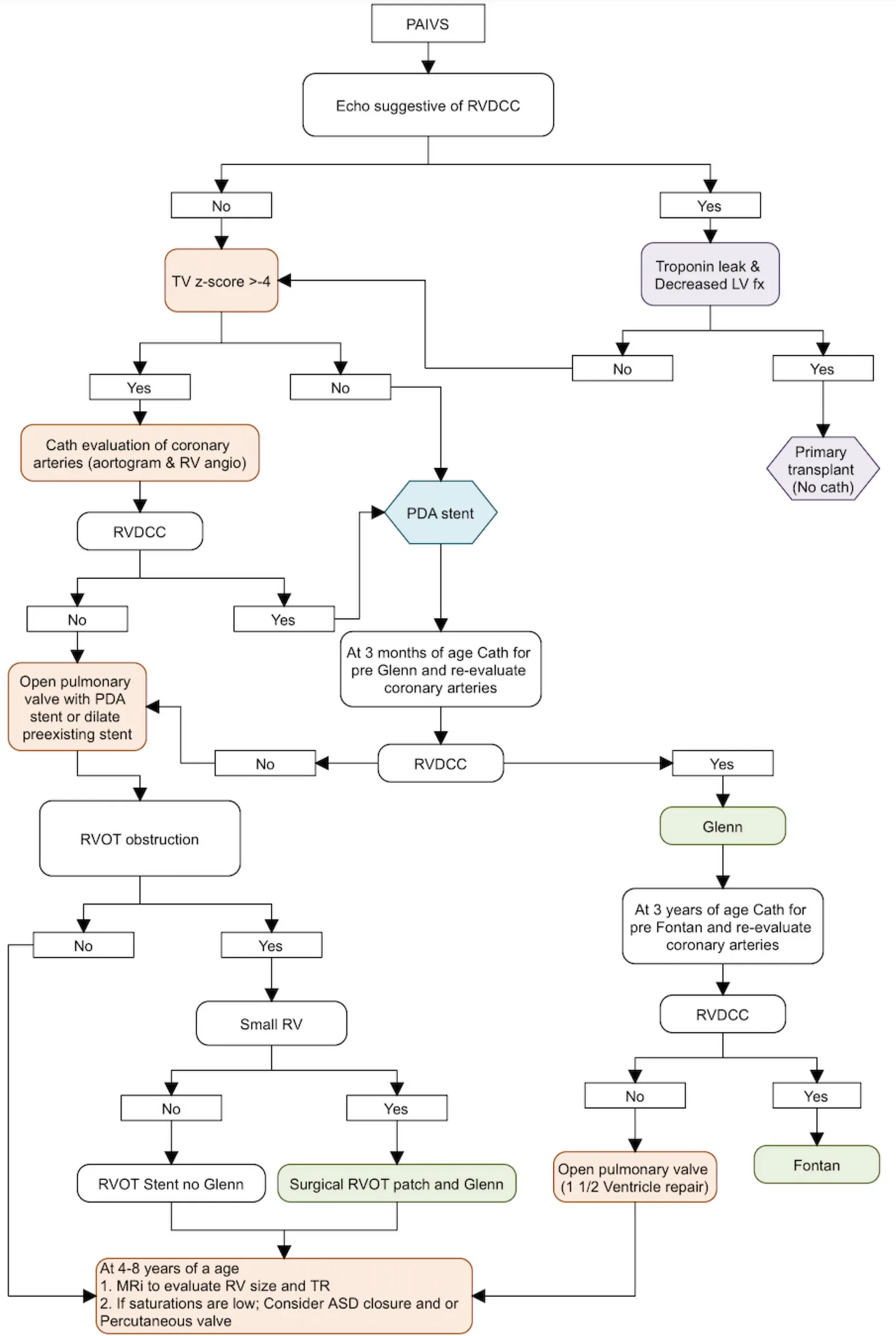
Post-hoc algorithm to help guide treatment strategy. *PA/IVS* pulmonary atresia intact ventricular septum, *RVDCC* right ventricular-dependent coronary circulation, *TV* tricuspid valve, *LV* left ventricle, *RV* right ventricle, *PDA* patent ductus arteriosus, *RVOT* right ventricular outflow tract, *TR* tricuspid regurgitation, *ASD* atrial septal defect

Our data also show low mortality in patients with PA/IVS. One patient underwent a neonatal transplant, and only 24.1% required single ventricle circulation, with 65.5% completing either a 2V or 1.5V circulation. Survival was 93.1%, with only two mortalities. We used our outcomes to establish a post-hoc algorithm to help guide treatment strategies for patients with PA/IVS (Fig. 4). If there is no echocardiographic evidence of RVDCC and the TV *z*-score is not diminutive, then patients will undergo cardiac catheterization to confirm the coronary findings and to proceed with pulmonary valvuloplasty. If, however, there is evidence of RVDCC, or if RVDCC cannot be definitively ruled out, or if there is a significantly diminutive TV *z*-score, then PDA stenting is done without RV decompression. This cohort of patients will then undergo a pre-Glenn cardiac catheterization at three months of age, where they will be re-evaluated for the presence of RVDCC. If, at that point, RVDCC can be ruled out, then patients can undergo pulmonary valvuloplasty. This process is repeated at three years of age during the pre-Fontan cardiac catheterization. Once again, if no RVDCC evidence is seen, patients will be diverted to a 1.5V circulation. This algorithm aims to maximize the possibility of avoiding 1V circulation by providing multiple checkpoints during which patients can be diverted away from this outcome. This is based on the recognition that there are circumstances where diagnosing RVDCC can be ambiguous. In these instances, we feel that it is safer to err on the side of caution by avoiding RV decompression and reassessing coronary anatomy at later points. Factors that can complicate the diagnosis of RVDCC early on include catheter-induced spasm that can lead to the appearance of severe stenosis, decreased flow in the presence of an angiogram with suboptimal filling, or competitive flow from a hypertensive RV in the presence of significant RV to coronary fistulas. In such instances where RVDCC cannot be ruled out, it is prudent to avoid RV decompression. We also recognize that decompressing all right ventricles that do not have RVDCC, regardless of TV z-score, can be a reasonable approach. However, that will require institutional and surgical buy-in.

## Limitation

Our study has multiple limitations. This is a single-center retrospective study, which limits the generalization of our results. In addition, our cohort is small, limiting the power of our analysis.

## Conclusion

Long-term survival following single ventricle circulation, especially in patients with pulmonary atresia with intact ventricular septum (PA/IVS), is complex and challenging. We introduce a post-hoc algorithm for patient risk stratification. This method promotes regular reassessments aimed at achieving the best possible survival rates with minimal morbidity and a high likelihood of complete or near complete septation.

## Data Availability

No datasets were generated or analysed during the current study.
